# Cytoprotective Effect of Antioxidant Pentapeptides from the Protein Hydrolysate of Swim Bladders of Miiuy Croaker (*Miichthys* *miiuy*) against H_2_O_2_-Mediated Human Umbilical Vein Endothelial Cell (HUVEC) Injury

**DOI:** 10.3390/ijms20215425

**Published:** 2019-10-31

**Authors:** Shi-Ying Cai, Yu-Mei Wang, Yu-Qin Zhao, Chang-Feng Chi, Bin Wang

**Affiliations:** 1 National and Provincial Joint Laboratory of Exploration and Utilization of Marine Aquatic Genetic Resources, National Engineering Research Center of Marine Facilities Aquaculture, School of Marine Science and Technology, Zhejiang Ocean University, Zhoushan 316022, China; shiyingcai1996@outlook.com; 2 Zhejiang Provincial Engineering Technology Research Center of Marine Biomedical Products, School of Food and Pharmacy, Zhejiang Ocean University, Zhoushan 316022, China; wangym731@126.com (Y.-M.W.); zhaoy@hotmail.com (Y.-Q.Z.)

**Keywords:** miiuy croaker (*Miichthysmiiuy*), swim bladder, FPYLRH, antioxidant activity, cytoprotective effect

## Abstract

In our previous research, ten antioxidant pentapeptides including FYKWP, FTGMD, GFEPY, YLPYA, FPPYERRQ, GFYAA, FSGLR, FPYLRH, VPDDD, and GIEWA were identified from the hydrolysate of miiuy croaker (*Miichthys* *miiuy*) swim bladder. In this work, their protective function on H_2_O_2_-induced oxidative damage to human umbilical vein endothelial cells (HUVECs) was studied. Results indicated that there was no significant difference in the HUVEC viability between the normal group and the treated groups with the 10 pentapeptides at the concentration of 100 μM for 24 h (*p* < 0.05). Furthermore, FPYLRH of 100 μg/mL extremely significantly (*p* < 0.001) increased the viability (80.58% ± 5.01%) of HUVECs with H_2_O_2_-induced oxidative damage compared with that of the model group. The protective mechanism indicated that FPYLRH could extremely significantly (*p* < 0.001) increase the levels of superoxide dismutase (SOD) (211.36 ± 8.29 U/mg prot) and GSH-Px (53.06 ± 2.34 U/mg prot) and decrease the contents of reactive oxygen species (ROS) (139.1 ± 11.8% of control), malondialdehyde (MDA) (13.66 ± 0.71 nM/mg), and nitric oxide (NO) (4.36 ± 0.32 µM/L) at the concentration of 100 μM in HUVECs with H_2_O_2_-induced oxidative damage compared with those of the model group. In addition, FPYLRH dose-dependently protected DNA in oxidative damage HUVECs model. These results suggested that FPYLRH could significantly attenuate the H_2_O_2_-induced stress injury in HUVECs and might be used as a potential natural antioxidant in the functional food industries.

## 1. Introduction

Reactive oxygen species (ROS) are essential components of the cells in organisms and play a dual role in the physiological processes involved in cell signaling and homeostasis [1,2]. Excessive ROS can cause oxidative damage to biomolecules in cell membranes, which further initiates a series of chronic diseases, such as rheumatoid arthritis, hypertension, diabetes, cardiovascular disease, atherosclerosis, chronic fatigue syndrome, and neurodegenerative diseases [1,3,4]. Retarding the progress of oxidative stress is a key step towards alleviating stress-related diseases [5]. In living organisms, antioxidant enzymes (superoxide dismutase (SOD), catalase (CAT), glutathione peroxidase (GSH-Px), and glutathione reductase (GSH-Rx)) and non-enzymatic antioxidant agents (glutathione (GSH), ascorbic acid, and β-carotene) can maintain the oxidative balance by reducing the concentration of ROS to prevent oxidative stress [2,6]. Therefore, the elimination of ROS damages by antioxidants is an effective treatment of those chronic diseases and food deterioration [7,8].

Recently, antioxidant peptides have been isolated and identified from various seafood proteins, such as edible marine fishes, alga, shellfish, and their processing by-products [3,9]. These peptides showed excellent properties, including high bioactivity, easy absorption, low molecular weight (MW), and lower toxicity or side effects, which are important for their potential application in health products [3]. Collagen peptides from jellyfish could alleviate ultraviolet (UV)-induced abnormal changes of antioxidant defense systems such as SOD and GSH-Px [10]. Similarly, Chen et al. found that gelatin hydrolysate of Pacific cod skin could prevent UV radiation-induced skin damage by suppressing the depletion of an endogenous antioxidant enzyme and the expression of nuclear factor-κB (NF-κB) and pro-inflammatory cytokines [11]. MDLFTE and WPPD from protein hydrolysate of *Tergillarca granosa* exhibited strong radical scavenging activities and high inhibiting ability on lipid peroxidation. In addition, MDLFTE and WPPD were stable and could retain strong antioxidant activity at the temperatures lower 80 °C and acidic and weakly alkaline environments (pH < 9) [12]. GPA from gelatin hydrolysate of fish skin showed an approximately 2.5-fold increase in antioxidant response element (ARE)-luciferase activity and suppressed the H_2_O_2_-induced intracellular ROS production by dose-dependently activating the expression of ARE-driven antioxidant enzyme genes [13]. The antioxidant functions of these peptides were thought to be associated with their MW, amino acid compositions and sequences, and spatial structures [3,14]. Moreover, these results suggested that seafood proteins were high-quality raw materials for the preparation of antioxidant peptides for protecting human health and food quality by reducing oxidative stress.

Miiuy croaker (*Miichthys miiuy*) is mainly spread from the Western Japan Sea to the East China Sea and has been extensively farmed in China since the late 1990s due to its high nutrient content, delicious taste, and economic value [14,15]. Previous research indicated that the swim bladder of miiuy croaker showed positive curative effects on dozens of diseases, such as protective liver function, removing ROS, cure dizziness, and warding against inflammation and cancers [1,16]. In our previous work, ten antioxidant pentapeptides were prepared from hydrolysate of miiuy croaker swim bladder and determined as FYKWP (S1), FTGMD (S2), GFEPY (S3), YLPYA (S4), FPPYERRQ (S5), GFYAA (S6), FSGLR (S7), FPYLRH (S8), VPDDD (S9), and GIEWA (S10) [1]. Of these, FPYLRH (S8) could effectively inhibit lipid peroxidation and exhibit strong scavenging activities on hydroxyl radical (EC_50_ 0.68 mg/mL), 2,2-diphenyl-1-picrylhydrazyl (DPPH) radical (EC_50_ 0.51 mg/mL), and superoxide anion radical (EC_50_ 0.34 mg/mL). In this work, the protective functions of the antioxidant pentapeptides, especially FPYLRH, regarding oxidative damage to human umbilical vein endothelial cells (HUVECs) by H_2_O_2_ were studied for further elucidating their antioxidant mechanism.

## 2. Results and Discussion

### 2.1. Effects of Antioxidant Pentapeptides (S1–S10) on the Viability of HUVEC

HUVEC is the main type of endothelial cell and provides a classic model system to study many aspects of endothelial function and disease, such as cell and cardiovascular protection effects of bioactive molecules, cardiovascular-related complications associated with various diseases, hypoxia and inflammation-related pathways in endothelia under normal and pathological conditions, etc. [17].

**Figure 1:**
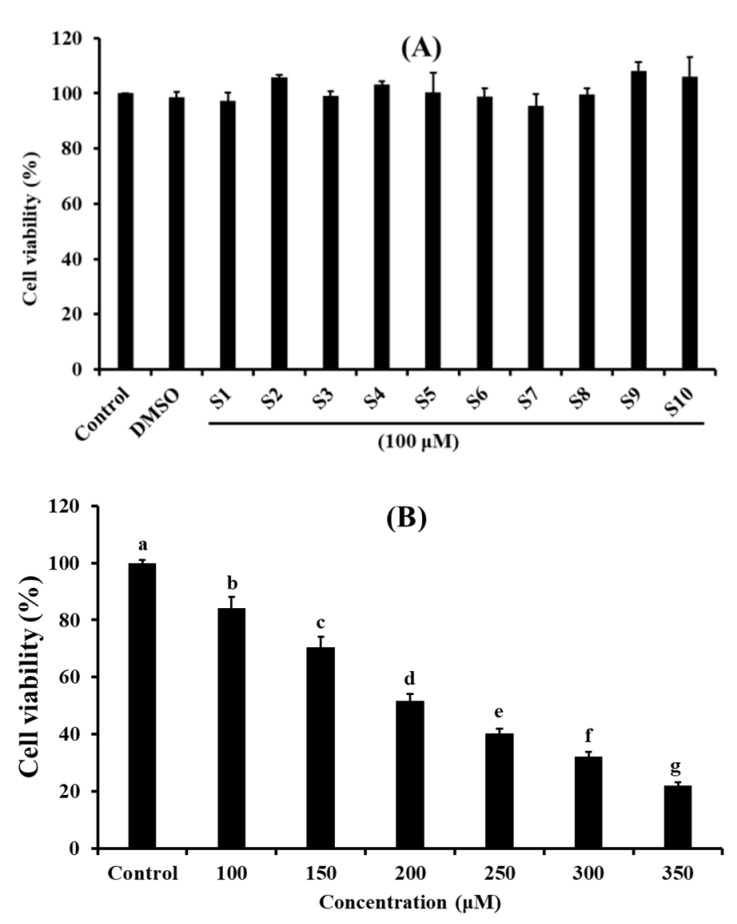
Effects of antioxidant pentapeptides S1–S10 at the concentration of 100 μM (**A**) and H_2_O_2_ with the concentration ranged from 100 to 350 μM (**B**) on cell viability of HUVECs for 24 h. Mean ± standard deviation (SD) (*n* = 3) is used to express the experiment data. ^a–g^ Values with same letter in Figure 1B indicate no significant difference of the cell viability of HUVECs treated with different H_2_O_2_ concentrations (*p* > 0.05).

As shown in Figure 1A, the viability of cells treated with FSGLR (S7) was 95.47% ± 4.20% at 100 μM for 24 h, which was lower than that of the other nine pentapeptides and normal control groups. The viability of cells treated with VPDDD (S9) was 108.20% ± 3.08%, which was higher than that of the other nine pentapeptides and normal control groups at the same concentration. However, there were no significant differences between the control group and peptides groups at 100 μM for 24 h (*p* < 0.05). Cell proliferation is a physiological process that occurs in almost all tissues and under many circumstances, and the balance between proliferation and programmed cell death (apoptosis) is maintained by regulating both processes to ensure the integrity of tissues and organs under normal conditions [18]. The cell viability measured by 3-(4,5-di methyl thiazol-2-yl)-2,5-diphenyltetrazolium bromide (MTT) method is an important index for evaluating the effects of pharmacological compounds on cell proliferation according to the response to stress stimuli, and it is often used to screen compounds for developing different pharmaceuticals [19]. Therefore, ten isolated pentapeptides (S1–S10) had the possibility for developing non-tumor functional products because of their insignificant effect on the normal proliferation of HUVECs.

Figure 1B indicated that the viability of HUVECs treated with H_2_O_2_ was negatively correlated with the concentrations ranging from 100 to 350 µM. In addition, the viability of HUVECs treated with H_2_O_2_ at the concentration of 200 µM was 51.66% ± 2.48%, which was significantly different from other groups (*p* < 0.05). Therefore, oxidative damage to HUVECs was established at the H_2_O_2_ concentration of 200 µM. Oxidative stress caused by ROS can activate apoptosis-related signaling pathways in vascular and cardiac endothelial cells, which further induce endothelial dysfunction to the initiation and development of cardiovascular diseases (CVDs) [20]. H_2_O_2_ can cause oxidative damage because it can be converted into hydroxyl radicals and oxygen radicals in liver cells [17]. Herein, a H_2_O_2_-induced HUVEC injury model was used to screen the antioxidant molecules and explore their mechanisms involved in the pathogenesis of ROS-induced oxidative stress.

### 2.2. Protective Effect of Antioxidant Pentapeptides (S1–S10) on the Oxidative Injury HUVEC by H_2_O_2_

**Figure 2:**
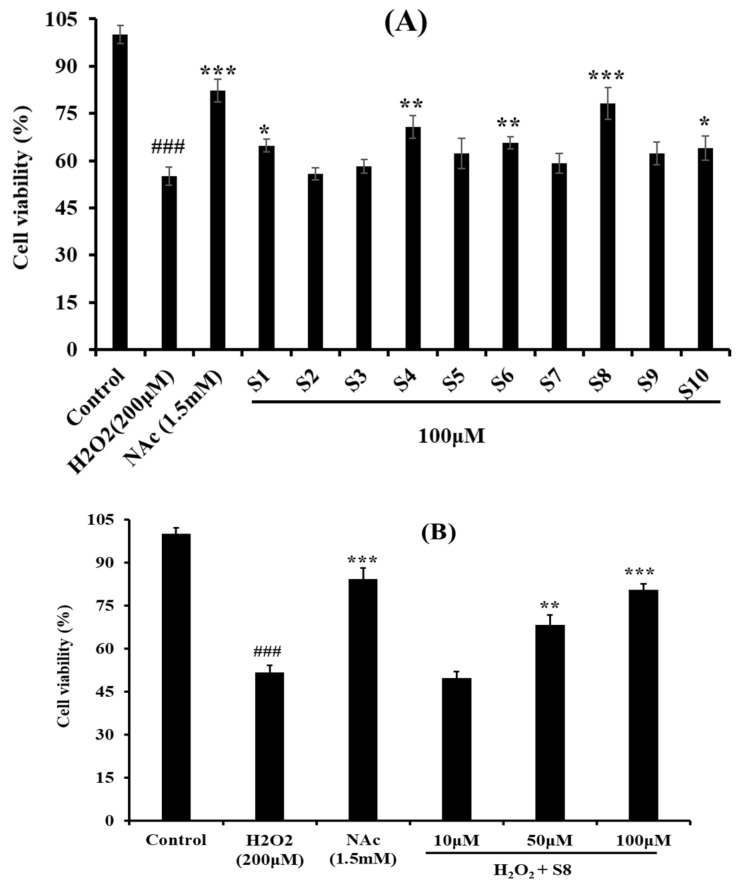
Protective effects of the 10 antioxidant pentapeptides (S1–S10) at the concentration of 100 μM (**A**) and FPYLRH (S8) at the concentrations of 10, 50, and 100 μM (**B**) on the H_2_O_2_-induced HUVEC injury model. Mean ± SD (*n* = 3) is used to express the experiment data. ^###^ *p* < 0.001 vs. Control group; *** *p* < 0.001, ** *p* < 0.01, and * *p* < 0.05 vs. H_2_O_2_-induced HUVEC injury model.

Figure 2A showed the protective effects of the 10 antioxidant pentapeptides (S1–S10) on the H_2_O_2_-induced HUVEC injury model, and the cell viabilities of the S1–S10-treated groups were improved compared with the model group. At the concentration of 100 μM, the HUVEC viability of the FPYLRH (S8)-treated group increased to 80.58% ± 5.01%, which was extremely (*p* < 0.001) significantly higher than that of the model group; the HUVEC viability of the YLPYA (S4)- and GFYAA (S6)-treated groups increased to 70.75% ± 3.65% and 65.67% ± 1.99%, respectively, which were very (*p* < 0.01) significantly higher than that of model group (*p* < 0.01); FYKWP (S1) and GIEWA (S10) increased the HUVEC viability to 64.74% ± 2.02% and 63.99% ± 3.78%, respectively, which were significantly (*p* < 0.05) higher than that of model group. The present data indicated that FPYLRH (S8) has the strongest protective ability on H_2_O_2_-induced oxidative damage HUVEC among the 10 antioxidant pentapeptides (S1–S10) at the concentration of 100 μM.

Figure 2B shows that there was a positive correlation between the concentration of FPYLRH (S8) and its protective ability on the H_2_O_2_-induced HUVEC injury model. The FPYLRH (S8)-treated group increased the HUVEC viability to 80.58% ± 5.01% and 68.21% ± 3.59% at the concentrations of 50 and 100 μM, respectively, which were extremely (*p* < 0.001) and very (*p* < 0.01) significantly higher than that of model group. However, there was no difference between the FPYLRH (S8) group at the concentration of 10 μM and the H_2_O_2_-induced oxidative damage group (*p* > 0.05).

**Figure 3:**
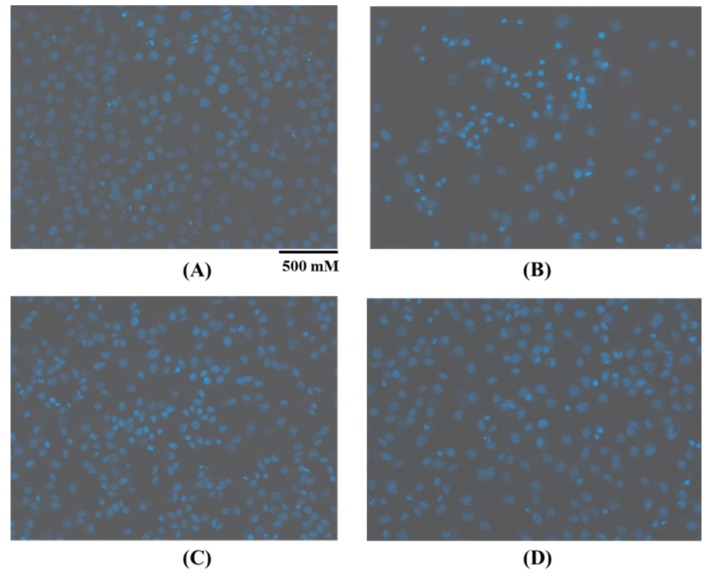
Apoptosis analysis of FPYLRH (S8) in the H_2_O_2_-induced HUVECs injury model at the concentration of 100 μM by Hoechst 33,342 staining assay. (**A**): Blank control; (**B**): Model (H_2_O_2_); (**C**): Positive control (acetylcysteine, NAc); (**D**): FPYLRH (S8).

Furthermore, images of Hoechst 33,342 staining HUVECs treated with H_2_O_2_, positive control of acetylcysteine (NAc), and FPYLRH (S8) at the concentrations of 100 μM for 24 h are shown in Figure 3. In the blank control group (Figure 3A), HUVECs were uniform in size, plump in shape, and presenting blue fluorescence. Compared with the blank control group, HUVECs in the model group (Figure 3B) showed a state of apoptosis because the number of HUVECs significantly decreased, the cells became smaller, and the fluorescence of most of the remaining adherent cells became bright. Figure 3C indicated that the most of HUVECs in the FPYLRH (S8) group adhered to the wall compared with the model group, and a small number of cells was washed away and another small number of cells showed blue fluorescence brightened compared with the blank control group. Those results indicated that FPYLRH (S8) had a strong protective effect on the oxidative damage HUVECs induced by H_2_O_2_, and the results were in line with those of Figure 2.

### 2.3. Effect of FPYLRH (S8) on the Levels of ROS in H_2_O_2_-Induced HUVEC Injury Model

**Figure 4:**
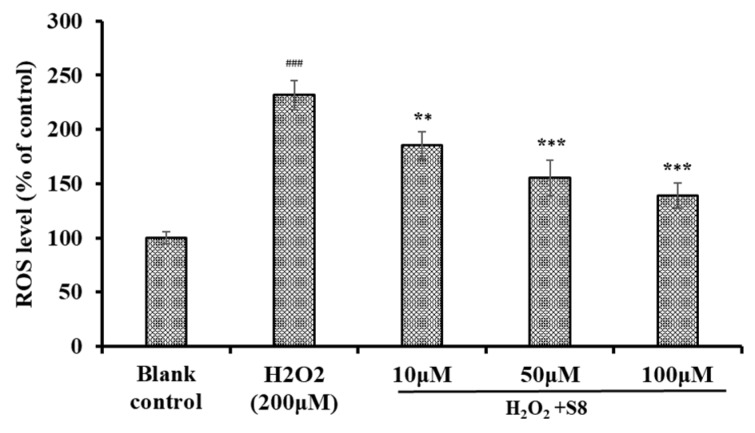
Effect of FPYLRH (S8) on ROS levels of H_2_O_2_-induced HUVEC injury model at the concentration of 10, 50, and 100 μM. Mean ± SD (*n* = 3) is used to express the experiment data. ^###^ *p* < 0.001 vs. Control group; *** *p* < 0.001 and ** *p* < 0.01 vs. H_2_O_2_-induced HUVEC injury model.

Excessive production of intracellular ROS will destroy key biological macromolecules, which further cause oxidative stress and serial chronic diseases [21,22]. Therefore, the effect of FPYLRH (S8) on the levels of ROS in HUVECs was measured. As shown in Figure 4, the level of ROS observed in the HUVECs exposed to H_2_O_2_ were 231.7% ± 13.5% of control, which was extremely (*p* < 0.001) significantly higher than those of the control group. As expected, the intracellular ROS levels (185.2% ± 12.4%, 155.3% ± 16.5%, and 139.1% ± 11.8% of control at the concentrations of 10, 50, and 100 μM) were significantly attenuated by FPYLRH (S8) pretreatment in a dose-effect manner.

### 2.4. Effect of FPYLRH (S8) on the Levels of GSH-Px, SOD, Malondialdehyde (MDA) and Nitric Oxide (NO) in H_2_O_2_-Induced HUVEC Injury Model

In living organisms, antioxidant enzymes (SOD, GSH-Px, CAT, and GSH-Rx) and non-enzymatic antioxidant agents (GSH, ascorbic acid, and β-carotene) make up a complex protective system, which can promote oxidative balance by reducing the concentration of ROS and forming less oxidation metabolites (MDA, NO, etc.) [6]. However, this protective system can be overwhelmed by uncontrolled generation of ROS, thus the system requires additional antioxidant agents to balance the oxidative status [6,19]. Therefore, effects on antioxidant enzymes and lipid oxidation metabolites in cells were usually applied to evaluate the antioxidant capacity of antioxidant molecules.

**Figure 5:**
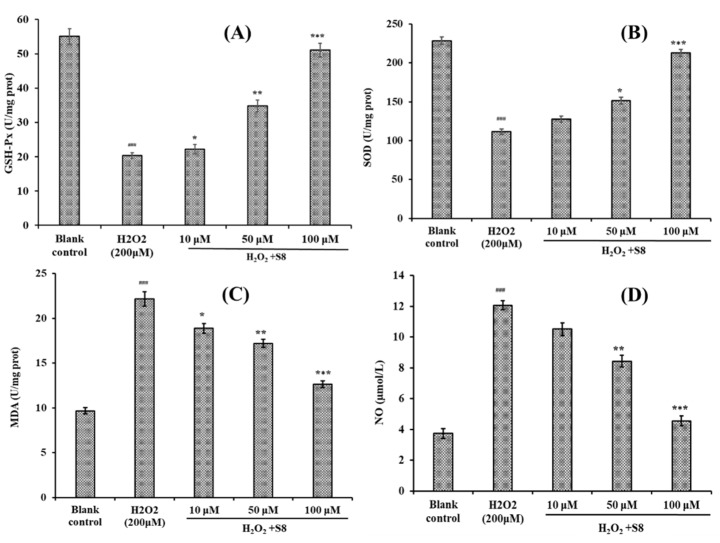
Effect of FPYLRH (S8) on GSH-Px (**A**), SOD (**B**), MDA (**C**), and NO (**D**) levels of the H_2_O_2_-induced HUVEC injury model at the concentrations of 10, 50, and 100 μM. Mean ± SD (*n* = 3) is used to express the experiment data. ^###^ *p* < 0.001 vs. control group; *** *p* < 0.001, ** *p* < 0.01, and * *p* < 0.05 vs. H_2_O_2_-induced HUVEC injury model.

As shown in Figure 5, the effects of FPYLRH (S8) on the levels of SOD, GSH-Px, MDA, and NO in HUVECs were measured for illuminating its protection in HUVECs with H_2_O_2_-induced oxidative damage. Figure 5A and Figure 5B indicated that the levels of GSH-Px and SOD observed in the HUVECs exposed to H_2_O_2_ were 20.38 ± 0.82 U/mg prot and 111.35 ± 3.47 mg prot, respectively, which were extremely significantly lower than those of the normal HUVECs control (*p* < 0.001). Moreover, the levels of GSH-Px (34.85 ± 1.65 U/mg prot and 51.06 ± 2.03 U/mg prot) and SOD (151.32 ± 4.52 U/mg prot and 212.56 ± 4.68 U/mg prot) of HUVECs incubated by FPYLRH (S8) at the concentrations of 50 and 100 μM were very (*p* < 0.01) and extremely (*p* < 0.001) significantly higher than those of the H_2_O_2_-damaged group.

Figure 5C,D showed that the contents of MDA and NO in the HUVECs exposed to H_2_O_2_ were 22.15 ± 0.81 nM/mg prot and 12.06 ± 0.29 µM/L, respectively, which were extremely significantly lower than those of the normal HUVECs control (*p* < 0.001). The data indicated that H_2_O_2_ at the concentration of 200 μM could significantly damage the membrane of HUVECs. Compared with the model group, the contents of MDA of HUVECs incubated by FPYLRH (S8) at the concentrations of 10, 50, and 100 μM were 18.89 ± 0.54 nM/mg prot, 17.21 ± 0.46 nM/mg prot, and 12.64 ± 0.38 nM/mg prot, respectively, which were significantly (*p* < 0.05), very significantly (*p* < 0.01) and extremely significantly (*p* < 0.001) lower than those of the model group. The contents of NO of HUVECs incubated with FPYLRH (S8) at the concentrations of 50 and 100 μM were 8.42 ± 0.38 µM/L and 4.56 ± 0.31 µM/L, respectively, which were very (*p* < 0.01) and extremely (*p* < 0.001) significantly lower than those of model group.

At present, some protein hydrolysates, fractions, and peptides showed intracellular ROS scavenging activities through regulating antioxidant enzyme levels [3]. Corn gluten peptide fractions of CPF1 (MW < 1 kDa) and CPF2 (1 < MW < 3 kDa) exhibited cytoprotective effects and intracellular ROS scavenging activities through increasing the activity levels of SOD, CAT, and GR) as well as the total GSH levels in oxidized HepG2 cells [23]. Liang, Zhang, and Lin reported that the pulse electric field (PEF) could increase the antioxidant activity of QDHCH from pine nut (*Pinus koraiensis*) [24]. Compared with H_2_O_2_ damaged group, the PEF-treated QDHCH has a better protective oxidative stress inhibition of 74.22% ± 3.70% by significantly increasing the T-SOD, CAT, GSH-Px, and GSH-Rx activities and decreasing the MDA content in HepG2 cells. Zheng et al. reported that GPA from fish skin gelatin hydrolysate could activate the expression of antioxidant response element (ARE)-driven antioxidant enzyme genes in a dose-dependent manner, and subsequently suppressed the H_2_O_2_-induced intracellular ROS production in IPEC-J2 cells [13]. EAMAPK and AVPYPQ from stracchino hydrolysate by in vitro gastro-intestinal digestion showing antioxidant activities in a wide concentration range (5–150 µg/mL), involving ROS reduction, SOD expression increases and Nrf2 antioxidant response activation in intestinal epithelial cells (IEC-6) [25]. The present results suggested that FPYLRH (S8) had similar cytoprotective functions with QDHCH, GPA, EAMAPK, and AVPYPQ through enhancing endogenous antioxidant defense systems to reduce the H_2_O_2_ damage in cells.

### 2.5. Protective Activity of FPYLRH (S8) on Oxidative Damage DNA Induced by H2O2

#### 2.5.1. Protective Activity on Plasmid DNA (pBR322 DNA)

**Figure 6:**
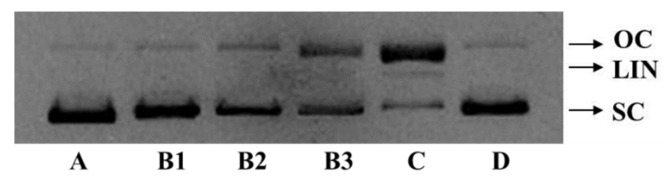
Agarose gel electrophoresis pattern of plasmid pBR322 DNA treated with H_2_O_2_ and FPYLRH (S8) at different conditions. A: pBR322 DNA; B1: pBR322 DNA + FeSO_4_ + FPYLRH (S8) (2.0 mg/mL) + H_2_O_2_; B2: pBR322 DNA + FeSO_4_ + FPYLRH (S8) (1.0 mg/mL) + H_2_O_2_; B3: pBR322 DNA + FeSO_4_ + FPYLRH (S8) (0.5 mg/mL) + H_2_O_2_; C: pBR322 DNA +FeSO_4_ + H_2_O_2_; D: pBR322 DNA + FeSO_4_ + GSH (2.0 mg/mL) + H_2_O_2_.

The excessive production of ROS may cause a quantity of degenerative processes in organisms, such as cancer, premature aging, cardiovascular, and neurodegenerative diseases, while DNA damage is a key step in these ROS-induced effects [19,26]. Therefore, the protective activity of FPYLRH (S8) on plasmid DNA (pBR322 DNA) oxidatively damaged by H_2_O_2_ is presented in Figure 6. The plasmid DNA (pBR322 DNA) was mainly of the supercoiled (SC) form under normal conditions (Figure 6A). The damage of plasmid DNA results in a cleavage of one of the phosphodiester chains and produces a relaxed open circular (OC) form. Further cleavage near the first breakage leads to linear (LIN) double stranded DNA molecules. The OC formation of DNA is indicative of single-strand breaks and the LIN formation of DNA is indicative of double-strand breaks [16].

In the experiment, hydroxyl radical was produced from iron-mediated decomposition of H_2_O_2_ when FeSO_4_ and H_2_O_2_ were added into and reacted in the sample solution, and it subsequently broke the plasmid DNA (pBR322 DNA) and converted the supercoiled form into the OC form (Figure 6C). The linear form of DNA was observed in Figure 6C, which indicated that the excess hydroxyl radicals further broke a small amount of the double-strand of DNA. As shown in Figure 6B, FPYLRH (S8) positively influenced the contents of SC form of the plasmid DNA (pBR322 DNA) in a dose-dependent manner. Correspondingly, the contents of OC form of the plasmid DNA (pBR322 DNA) were decreased with increasing concentration of FPYLRH (S8). Moreover, FPYLRH (S8) at the concentration of 2 mg/mL showed similar protective effect on DNA damage with the positive control of GSH (Figure 6D). Therefore, FPYLRH (S8) could protect the supercoiled plasmid DNA through preventing the reaction of Fe^2+^ with H_2_O_2_ and scavenge hydroxyl radical by donating a hydrogen-atom or electron. This finding was in line with the result that FPYLRH (S8) could effectively scavenge hydroxyl radical in radical scavenging assay in vitro [1].

#### 2.5.2. Protective Activity on DNA in H_2_O_2_-Induced HUVEC Injury Model

The comet assay, known as single cell gel electrophoresis (SCRE), helps to determine whether there has been DNA damage to a single cell from apoptosis (cell death) or cytotoxicity and the extent of this damage [27]. Cells embedded in agarose on a microscope slide are lysed with detergent and high salt to form nucleoids containing supercoiled loops of DNA linked to the nuclear matrix. Electrophoresis results in structures that resemble comets at high pH and the intensity of the comet tail relative to the head that reflects the number of DNA breaks [28]. The comet assay has been devoted to testing novel chemicals for genotoxicity, monitoring environmental contamination with genotoxins, human biomonitoring and molecular epidemiology, and basic research in DNA damage and repair [28,29].

**Figure 7:**
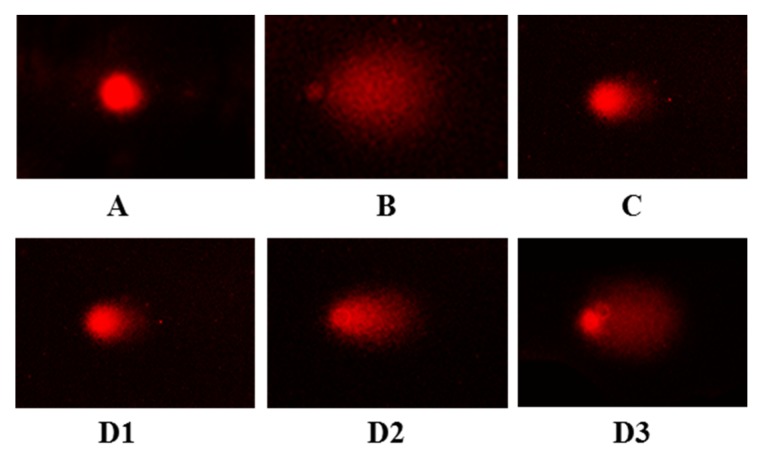
Typical comet images observed by PI (Propidium iodide) staining. (**A**): Blank control group; (**B**): Model (H_2_O_2_, 200 µM) group; (**C**): Positive control (NAc) group; (**D1**): H_2_O_2_ + FPYLRH (S8) (200 µM); (**D2**): H_2_O_2_ + FPYLRH (S8) (100 µM); (**D3**): H_2_O_2_ + FPYLRH (S8) (50 µM).

As shown in Figure 7A, the comet head is bright and almost no comet tail is found in the blank group, but the comet tail is long and has a large area in the model group (Figure 7B), which indicated that the damage model was successfully established. Compared with the model group, the comet tails on damaged HUVECs were gradually reduced with the increasing concentration of FPYLRH (S8) (Figure 7D). In addition, the length and area of comet tails in FPYLRH (S8) group with the concentration of 200 µM was close to that of positive control group (Figure 7C).

The comet tail length or comet length can intuitively reflect the experimental results, but they usually cause large errors. For a more accurate expression of the data of the comet assay, more indicators including head DNA (HDNA), tail DNA (TDNA), and torque class indicator (Olive tail moment, OTM) were employed (Table 1). The results indicated that the TDNA, tail length (TL), tail moment (TM), and OTM of model group were extremely significantly larger than that of the blank control group (*p* < 0.001), the comet length (CL) of model group was significantly larger than that of blank control group (*p* < 0.05), and HDNA was extremely significantly less than that of the blank control group (*p* < 0.001). With the decrease of the concentration of FPYLRH (S8), the HDNA of comet decreased gradually, while the TDNA, CL, TL, TM, and OTM increased gradually. Moreover, there were extremely significant differences of measured indicators at tested concentrations, except CL at 50 µM, between the sample and model groups (*p* < 0.001). Therefore, the comet assay indicated that FPYLRH (S8) showed a significant protective effect on DNA in H_2_O_2_-induced HUVEC injury model.

Oxidative damage DNA leads to pathological processes involved in the development of cancer, cardiovascular diseases, and ageing [30], and the damage degree of DNA can increase under conditions of oxidative stress, arising from exposure to a variety of physical or chemical insults [31,32]. Antioxidant peptides attract extensive attention due to their key roles in maintaining cellular function upon DNA insult. Zhao et al. indicated that a peptide fraction from croceine croaker swim bladder could have an anti-fatigue effect through inhibiting the oxidative reactions and DNA damage [16]. I/LNI/LCCN and WCTSVS from marine *Sepia brevimana* and *Loligo duvauceli*, respectively, exhibited significant protective effects on DNA damage and inhibition for the linoleic acid auto-oxidation in the model system due to hydroxyl radical induction [33]. YGDEY protected liver hepatocellular cells (HepG2 cells) from alcohol-induced injury by inhibiting oxidative stress including decreasing the amount of ethanol-induced DNA damage, and this may be associated with the Akt/NF-κB/mitogen-activated protein kinase (MAPK) signal transduction pathway [34]. In our previous research, FPYLRH (S8) exhibited strong radical scavenging activity and lipid peroxidation inhibition [1], and the presented results indicated FPYLRH could decrease the H_2_O_2_-induced stress injury in HUVECs by increasing the levels of SOD and GSH-Px, decreasing the contents of MDA and NO, and protecting DNA in oxidative damage. The antioxidant mechanism of FPYLRH might be related to activating the Nrf2-antioxidant response element signaling pathway.

### 2.6. Relationship Among the Molecular Size, Amino Acid Composition, and Antioxidant Activity

Molecular size and amino acid composition significantly affect the biological function of peptides [3,35]. Short peptides with 2–10 amino acid residues showed high radical scavenging and inhibition activities of lipid peroxidation because they were more easily accessible to active radicals to provide potential effects in a reaction mixture than their parent native proteins [36,37]. In the experiment, FPYLRH (S8) exhibited strong cytoprotective effect on H_2_O_2_-mediated HUVECs, which indicated that FPYLRH (S8) with 6 amino acid residues could easily enter into cells, interact with ROS, and inhibit oxidative stress reaction.

Hydrophobic amino acids with non-polar aliphatic groups, such as Tyr, Leu, Trp, Pro, Ile, and Val, had high reactivity to hydrophobic PUFAs and exert their significant effects on radical scavenging [1,38]. Aromatic amino acids (Phe, Trp, and Tyr) with aromatic residues can donate protons to electron-deficient radicals, keeping ROS stable during the radical scavenging process [14,17]. Sheih et al. reported that Tyr residues could remove free radicals, change them into more stable phenoxy radicals, and further inhibit the peroxidizing chain reaction mediated by free radicals [39]. Chen et al. found that the pyrrolidine ring of Pro can increase the flexibility of peptides and quench singlet oxygen due to its low ionization potential [40]. Therefore, the hydrophobic amino acid residues (Leu and Pro) and the aromatic residues (Phe and Tyr) in the sequences of FPYLRH (S8) should contribute to its radical-scavenging and lipid peroxidation inhibitory activities. In addition, polar amino acids are reported to play a critical role in the metal ion chelating and hydroxyl radical scavenging activities, which is related to the carboxyl and amino groups in their side chains [3,41,42]. Memarpoor-Yazdi et al. found that the basic (Arg) amino acid residues in the sequence of NTDGSTDYGILQINSR were of great importance to their antioxidant activities [43]. Therefore, the presence of Arg and His in FPYLRH (S8) should play a crucial role for their antioxidant capabilities.

## 3. Method and Materials

### 3.1. Materials

HUVECs were purchased from the Cell Bank of Type Culture Collection of the Chinese Academy of Sciences (Shanghai, China). Dulbecco’s modified Eagle’s medium (DMEM), phosphate buffered saline (PBS, pH 7.2), NAc, dimethyl sulfoxide (DMSO), 3-(4,5-dimethylthiazol-2-y1)-2,5-diphenyltetrazolium bromide (MTT) were purchased from Sigma-Aldrich (Shanghai) Trading Co., Ltd. (China). FYKWP (S1), FTGMD (S2), GFEPY (S3), YLPYA (S4), FPPYERRQ (S5), GFYAA (S6), FSGLR (S7), FPYLRH (S8), VPDDD (S9), and GIEWA (S10) with a purity higher than 98% were synthesized in China Peptides Co. (Suzhou, China).

### 3.2. Cell Culture and Viability Assay

The cell culture method and viability assay were performed according to the method of Lim et al. [44]. The HUVECs were cultured in DMEM containing 1% penicillin-streptomycin and 10% FBS at 37 °C and 5% CO_2_ atmosphere. The MTT test was used to measure the cell viability. After 24 h incubation in a 96-well plate (7 × 10^3^ cells/well), the HUVECs were cultured in 100 μM peptide solution for 12 h. After that, the wells were washed twice with PBS and the MTT with a final concentration of 0.5 mg/mL was added for an additional 4 h. After that, the formazan crystals formed by active cells were dissolved in 150 µL of DMSO. The absorbance at 570 nm was measured and the cell viability was calculated by the following equation:

Cell viability = (A_sample_/A_control_) × 100%. (1)

### 3.3. Protection of FPYLRH on Oxidative Damage HUVECs by H_2_O_2_

The HUVECs were seeded on a 96-well plate with the density of 1.5 × 10^4^ cells/well. After culturing for 24 h, the supernatant was aspirated and H_2_O_2_ (final concentration of 100, 200, 300, 400, and 500 mM) was added and sequentially incubated for 24 h. The optimal H_2_O_2_ concentration was confirmed when the cell viability was close to 50%.

The HUVECs with oxidative damage under the H_2_O_2_-induced optimal conditions were used to test the protective effects of FPYLRH on the damaged cells. The peptides were dissolved in the DMEM medium with the concentrations of 10, 50, and 100 µg/mL. The selected HUVECs were cultured (1.5 × 10^4^ cells/well) in a 96-well plate for 24 h. Then the supernatant was aspirated and 100 µL of samples were added into the protection groups respectively incubating for 8 h. After removing samples, H_2_O_2_ was added into the damage and protection groups with the optimal concentration and sequentially incubated for 24 h. The positive control group used 100 µL of 1.5 mM NAc instead of 100 µL of peptide solution.

### 3.4. Hoechst 33,342 Staining Assay

The logarithmic growth HUVECs were treated by trypsinization and grown (2.0 × 10^5^ cells/well) in a 6-well plate for 24 h. Then the supernatant was aspirated and 300 µL of FPYLRH and NAc solutions were separately added into the protection and positive control groups incubating for 2 h, respectively. After removing samples, 300 µL of H_2_O_2_ was separately added into the model, protection, and positive control groups which were incubated for 24 h, respectively. After that, HUVECs were exposed to 8 mg/mL Hoechst 33,342 solution for 30 min at 37 °C and 5% CO_2_ atmosphere. After removing the Hoechst 33,342 solution and washing three times with serum-free DMEM, the morphology of HUVECs was observed using a fluorescence microscope (LSM710; Carl Zeiss Microscopy GmbH, Jena, Germany) with the excitation and emission wavelengths of 550 and 460 nm, respectively.

### 3.5. Determination of the Levels of ROS in H_2_O_2_-Induced HUVECs

Intracellular ROS accumulation in HUVECs was monitored according to the previous method described by Zheng et al. [13]. In brief, HUVECs were preincubated with samples at the concentrations of 10, 50, or 100 μM (B) for 12.0 h, and then incubated with 200 μM H_2_O_2_ for 2 h. After that, the cells were washed with PBS and incubated with 10 μM DCFH2-DA in fresh culture medium for 0.5 h. Intracellular ROS levels indicated by DCF fluorescence were quantified on a BD FACS Calibur flow cytometer (BD Biosciences, San Diego, C, USA) using excitation and emission filters of 488 and 530 nm, respectively. The data were expressed as % of control values.

### 3.6. Determination of the Levels of Antioxidant Enzymes in H_2_O_2_-Induced HUVECs

HUVECs were cultured in 6-well plates (1 × 10^6^ cells/well). FPYLRH with final concentration of 10, 25, and 50 µg/mL was added into the protection groups, respectively. Last, the damage and protection groups were exposed to H_2_O_2_. Subsequently, 500 mL of cell lysis buffer was added into each well on ice lysed for 30 min and centrifuged at 12,000× *g* and 4 °C for 10 min. The resulting liquid supernatant was subsequently stored on cold standby at 4 °C (the indicators should be measured within 6 h). The levels of SOD, GSH-Px, NO, and MDA were measured using assay kits according to the protocols of the manufacturer (Nanjing Jiancheng Bioengineering Institute Co., Ltd., Nanjing, China), and protein concentrations were determined using the bicinchoninic acid (BCA) method to normalize their levels. The levels of T-SOD and GSH-Px were expressed as units of enzymatic activity per milligram of protein (U/mg prot).

### 3.7. Protective Effect of FPYLRH on pBR322 Plasmid DNA Damaged by H_2_O_2_

The ability of FPYLRH to protect supercoiled pBR322 plasmid DNA was measured according to the previous method [16]. The reaction mixtures (15 µL) containing 5 µL of PBS (10 mM, pH 7.4), 1µL of plasmid DNA (0.5 µg), 5 µL of FPYLRH, 2 µL of 1 mM FeSO_4_, and 2 µL of 1 mM H_2_O_2_ were incubated at 37 °C for 30 min. After incubation, 2 µL of loading buffer (50% glycerol (*v*/*v*), 40 mM EDTA, and 0.05% bromophenol blue) was added to stop the reaction, and the reaction mixtures were electrophoresed on 1% agarose gel containing 0.5 µg/mL ethidium bromide in Tris/acetate/EDTA gel buffer for 50 min (60 V). The DNA in the gel was visualized and photographed under ultraviolet light. GSH was used as positive control.

### 3.8. DNA Comet Assay

The DNA comet assay was according to the previous method [45]. One hundred μL of 0.5% normal melting point agarose (NMA) preheated at 45 °C was spread on the frosted glass slides at 45 °C and covered with a clean cover glass. Subsequently, the slide was placed at 4 °C allowing the NMA to set. After 10 min, the slide was taken out, and the cover glass was gently peeled off. Then 75 μL melted 0.7% NMA mixed with 10 μL cells (1 × 10^6^ cells/mL) were quickly dropped to the first layer of gel and immediately covered with another clean cover glass. Then, the slide was placed at 4 °C for 10 min allowing the NMA to set. After removing the cover glass slide, the glass slide was placed in the plate, the pre-cooled lysis buffer was added, and the plate was put into a refrigerator at 4 °C for 1 h. The glass slide was taken out, rinsed with PBS, and placed in an electrophoresis tank in cold 0.3 M NaOH, 1 mM EDTA for 20–60 min. After that, the electrophoresis was carried out for 25 min at a constant voltage of 25 V. After electrophoresis, the gel was neutralized with 0.4 mM Tris-HCl buffer (pH 7.5) at 4 °C for 10 min, and the neutralization process was repeated three times. Gels were stained with PI (propidium iodide) dye liquor for 10 min and measured with a fluorescence microscope at 515~560 nm wavelength. About 100 cells per gel were randomly selected for measuring the diameter and migration length of DNA.

### 3.9. Statistical Analysis

Data were presented as means ± standard deviation (SD) (*n* = 3). ANOVA test (SPSS 19.0 software) was applied to compare the mean value of each treatment. Significant differences were determined by using Duncan’s multiple range Test (*p* < 0.05).

## 4. Conclusions

In this work, the protective effect of 10 antioxidant pentapeptides (S1–S10), especially FPYLRH (S8), from the hydrolysate of miiuy croaker swim bladder on oxidative damage of HUVECs by H_2_O_2_ was studied systematically. There was no significant difference between the normal HUVECs and the HUVECs treated with the 10 pentapeptides at 100 μM for 24 h (*p* < 0.05). Furthermore, FPYLRH could significantly inhibit H_2_O_2_-induced oxidative stress in HUVECs compared with that of the model group through increasing the levels of SOD and GSH-Px, decreasing the contents of ROS, MDA, and NO, and protecting DNA in HUVECs with oxidative damage compared with the model group. These results suggested that FPYLRH could be used as a potential natural antioxidant in functional food and other products.

## Figures and Tables

**Table 1:** Protective activity of FPYLRH (S8) on DNA in H_2_O_2_-induced HUVEC injury model in comet assay.

Group	Control	Model	NAc	FPYLRH (S8, µM)		
				200	100	50
Cell Number (n)	104	117	121	111	107	118
HDNA (%)	91.9 ± 4.2	18.2 ± 3.2 ^###^	80.7 ± 3.8 ***	78.4 ± 7.9 ***	53.2 ± 6.2 ***	37.5 ± 5.1 ***
TDNA (%)	8.1 ± 4.2	81.8 ± 3.2 ^###^	19.3 ± 4.8 *	21.6 ± 7.9 ***	46.8 ± 6.2 ***	62.5 ± 5.1 ***
CL (pix)	68.3 ± 3.3	77.3 ± 13.5 ^#^	66.2 ± 3.3 ***	58.4 ± 5.7 ***	60.0 ± 7.3 ***	76.1 ± 10.6 *
TL (pix)	7.3 ± 0.33	59.3 ± 6.6 ^###^	27.2 ± 1.6 ***	23.4 ± 2.4 ***	25.0 ± 3.3 ***	45.1 ± 6.5 ***
TM	0.6 ± 0.03	46.4 ± 8.1 ^###^	5.2 ± 0.24 ***	5.0 ± 0.5 ***	11.7 ± 1.4 ***	28.1 ± 3.9 ***
OTM	2.3 ± 0.1	24.5 ± 4.3 ^###^	4.7 ± 0.2 ***	4.7 ± 0.5 ***	10.4 ± 1.2 ***	17.2 ± 2.4 ***

Head DNA, tail DNA, comet length, tail length, tail moment, and olive tail moment are referred to as HDNA, TDNA, CL, TL, TM, and OTM, respectively. All data are presented as the mean ± SD of triplicate results. ^#^ *p* < 0.05 vs. Control group, ^###^ *p* < 0.001 vs. Control group; * *p* < 0.05 vs. model group, *** *p* < 0.001 vs. model group.
